# Supplementary material for: Electrophysiological signatures of spelling sensitivity development from primary school age to adulthood
Source: Sci Rep. 2024 Mar 30;14:7585. doi: 10.1038/s41598-024-58219-z (PMC10981698; doi:10.1038/s41598-024-58219-z)
Supplement: Supplementary file 2 — Supplementary Information 2. [file 41598_2024_58219_MOESM2_ESM.docx]

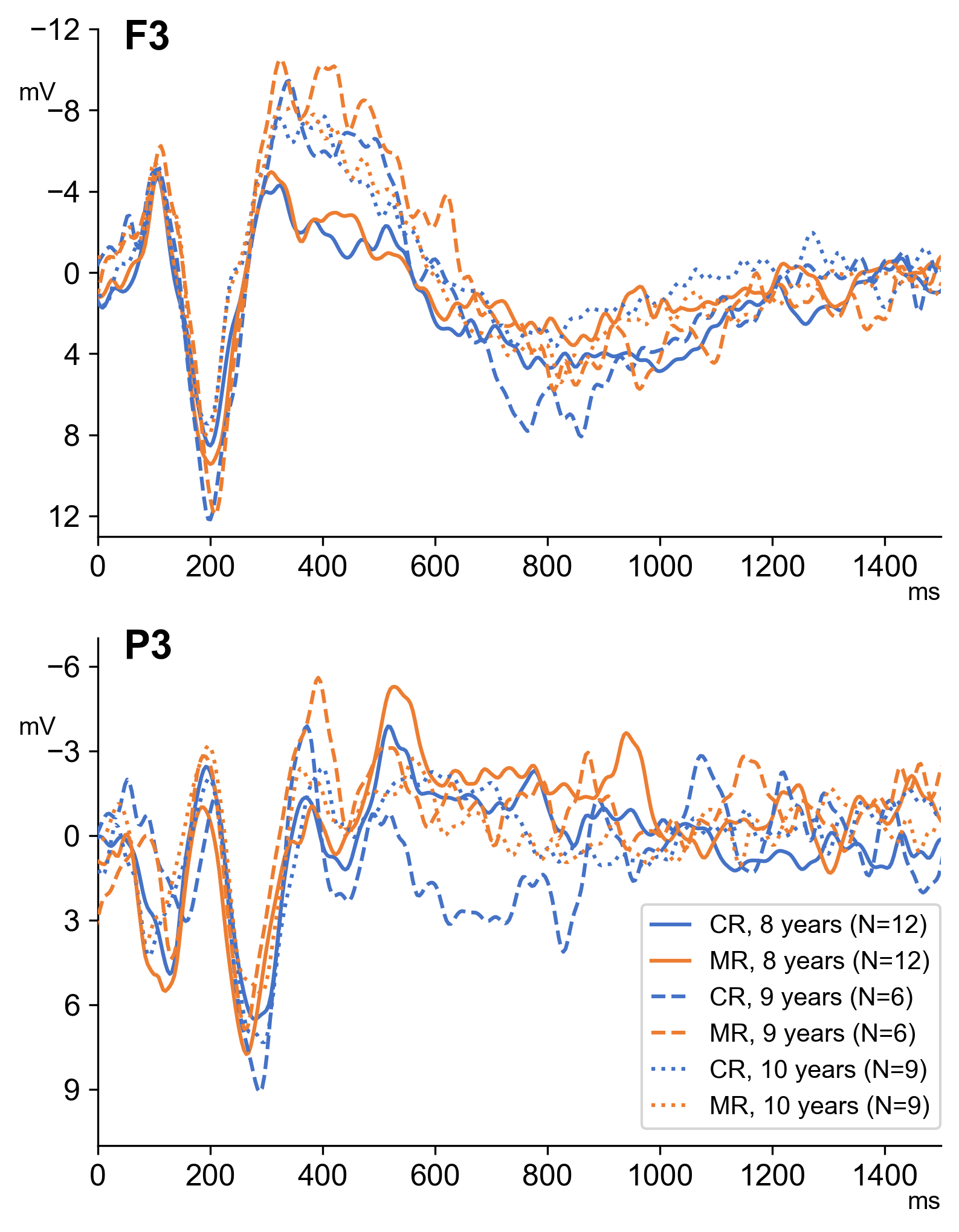


The figure shows the averaged ERPs for correct (CR) and misspelling (MR) words in children aged 8, 9, and 10 for P3 and F3. We did not find any difference between ERPs to CR and MR conditions in these subgroups of children.
